# Intermediate results of implementation of automatic electronic alert program for early detection of severe sepsis patients in an hospital with sepsis unit. analysis of two years period

**DOI:** 10.1186/2197-425X-3-S1-A218

**Published:** 2015-10-01

**Authors:** R Zaragoza, S Sancho, C Hurtado, J Camarena, R González, S Borrás, M Cervera

**Affiliations:** Hospital Universitario Dr. Peset, Valencia, Spain

## Intr

Sepsis is a time-dependent process that must be early detected and treated. Automatic alerts may play an important role in prompt detection.

## Objectives

To describe the clinical and epidemiological characteristics of patients with severe sepsis and septic shock detected by a sepsis unit in ED of a tertiary hospital and to analyse the influence of the IMPLEMENTATION OF AUTOMATIC ELECTRONIC ALERT PROGRAM (AEAP) on process indicators, length of stay and outcome of patients with severe sepsis and septic shock detected by a sepsis unit.

## Methods

During an two years period, severe sepsis and septic shock patients detected in a teaching hospital were prospectively evaluated, Clinical and microbiological variables and process indicators such as delay of lactate extraction, inadequate empirical antibiotic treatment (IEAT) rates and antibiotic administration were recorded. Mortality rates, % of admissions at ICU and length of stay were also collected. Two different periods were analysed in order to analyse the possible differences in process indicators, mortality rates and length of stay. Period A: From 1-October-2102 to 15-June-2013 when a manual electronic check list to guide the detection of these patients was applied and Period B: From 16-June-2013 to 30-September 2014 when AEAP was implemented. A univariate analysis was performed to define the possible differences between to periods using SPSS package (15.0). Statistical significance was considered when p value < 0.05.

## Results

1039 cases of severe sepsis and septic shock were detected (34.1% septic shock). Mean age was 72 ± 15 years, APACHE II and SOFA score were 17.7 ± 6.5 and 5 ± 3 respectively. The primary focus of infection was the respiratory (42.5%) followed by urinary (25.4%). Global mortality was 23.3%. Antibiotic administration was performed at 161.9 ± 179.1 min. in period A and 153.74 ± 156.49 min. at period B (p = 0.25). No differences in delay of lactate extraction were found (86,0 ± 112,7 vs 81,32 ± 191,4 min). The number of activations was higher in period B (254 vs. 785 episodes) however the rate of admissions in ICU diminished in a significant way. (30.7 % vs. 27.4%; p = 0.03) Statistically significant differences were also observed for the rate of appropriate empirical antibiotic therapy (40% vs. 13%. p = 0.01) between two periods. Although there were no significant differences in global mortality rate (23.2% vs. 23.3%) and length of stay (11.8 ± 13,6 vs 10.60 ± 13,7 days) between two groups when patients with LTS were excluded the diminution of mortality rates became significant (14.6% vs. 13.7%; p = 0,02).

## Conclusions

These intermediate results showed an clearly benefit of AEAP in terms of detection, diminution of admissions in ICU and mortality rates in patients without LTS without any changes in process indicators. A significant reduction of inappropriate empirical antibiotic therapy may be the cause of these improved results.

